# Urbanization and Waterborne Pathogen Emergence in Low-Income Countries: Where and How to Conduct Surveys?

**DOI:** 10.3390/ijerph17020480

**Published:** 2020-01-11

**Authors:** Alexandra Bastaraud, Philippe Cecchi, Pascal Handschumacher, Mathias Altmann, Ronan Jambou

**Affiliations:** 1 Laboratoire d’Hygiène des Aliments et de l’Environnement, Institut Pasteur de Madagascar, BP 1274, Antananarivo 101, Madagascar; abastaraud@pasteur.mg; 2 MARBEC (IRD, IFREMER, UM2 and CNRS), University Montpellier, 34095 Montpellier, France; philippe.cecchi@ird.fr; 3 Centre de Recherche Océanologique (CRO), Abidjan BPV 18, Ivory Coast; 4 IRD UMR 912 SESSTIM, INSERM-IRD-Université de Marseille II, 13000 Marseille, France; p.handschumacher@unistra.fr; 5 ISPED Université Victor Segalen Bordeaux II, 146 rue Leo Saignat, 33076 Bordeaux cedex, France; mathias.altmann@u-bordeaux.fr; 6 Département de Parasitologie et des insectes vecteurs, Institut Pasteur Paris, 75015 Paris, France

**Keywords:** waterborne diseases, drug resistance, urbanization, surface water, plastics, metagenomic

## Abstract

A major forthcoming sanitary issue concerns the apparition and spreading of drug-resistant microorganisms, potentially threatening millions of humans. In low-income countries, polluted urban runoff and open sewage channels are major sources of microbes. These microbes join natural microbial communities in aquatic ecosystems already impacted by various chemicals, including antibiotics. These composite microbial communities must adapt to survive in such hostile conditions, sometimes promoting the selection of antibiotic-resistant microbial strains by gene transfer. The low probability of exchanges between planktonic microorganisms within the water column may be significantly improved if their contact was facilitated by particular meeting places. This could be specifically the case within biofilms that develop on the surface of the myriads of floating macroplastics increasingly polluting urban tropical surface waters. Moreover, as uncultivable bacterial strains could be involved, analyses of the microbial communities in their whole have to be performed. This means that new-omic technologies must be routinely implemented in low- and middle-income countries to detect the appearance of resistance genes in microbial ecosystems, especially when considering the new ‘plastic context.’ We summarize the related current knowledge in this short review paper to anticipate new strategies for monitoring and surveying microbial communities.

## 1. An Urgent Need to Investigate Environmental, Human, and Animal Microbiota Interactions

**Figure 1:**
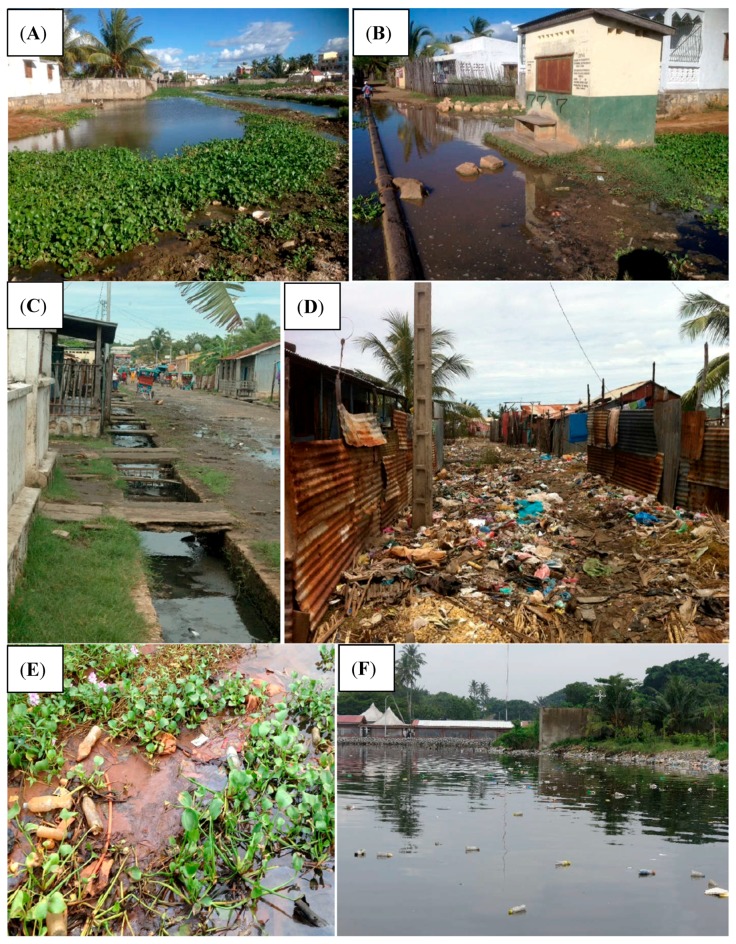
Plastics and garbage in an open sewer network; (**A**–**D**) Mahajanga, Madagascar, 2014, (**A,B**) open sewer network, (C) standpipe in a flooded area, (D) garbage pen in an area liable to flooding; (**E**,**F**) Ébrié Lagoon in Abidjan, floating plastics.

The United Nations predicts that half of the population in Africa and Asia will be urban by 2030 and that the African population will double (to 2.4 billion) by 2050. This global increase in population sustains a growing rate of five million residents per month in towns in low- and middle-income countries. This increase is essentially due to newborns but also to migration from rural areas. Urbanization presents opportunities and challenges for poverty reduction [1], but this rapid urbanization coupled with inadequate urban planning will disturb the functional organization of the cities [2]. The increase in populations in Africa induces “slumization” in towns with informal settlements and limited access to good-quality drinking water and sanitation facilities [3]. The arrival of migrants from rural areas also sustains the transfer of animals and environmental strains of bacteria, which mix with urban “microbiota.” In suburbs, surface water is often contaminated by animal and human feces from septic tanks or sewage discharges [4,5,6,7,8,9] (Figure 1). In harbors, the surface water can even be contaminated with ballast water from ships, transferring microorganisms from one part of the world to another [10,11]. In towns, all these microbial communities will mix in a large environmental cauldron favoring genetic exchanges. At the same time, these microbial communities will be exposed to antibiotics or chemicals released into surface water with urban wastewater, favoring the selection of new resistant strains. In towns, surface water can become highly contaminated. There are 828 million people living in slums over the world, which includes 62% of the urban population of sub-Saharan Africa and 43% of the urban population of South-Central Asia [12,13]. An estimated 141 million citizens have no access to good-quality drinking water, and 794 million have no access to sanitation facilities. Unfortunately, defects in the water supply in towns or suburbs (i.e., ineffective water treatment, frequent shortages, or low pressure) favor the direct use of these surface waters by these inhabitants for domestic uses, resulting in a high incidence of waterborne diseases. This threat mainly concerns children, noticeably in sub-Saharan African countries (see Figure 12 in Landrigan et al., 2018 [14]). Urban farming using surface water or fishing in polluted water (e.g., the Ébrié Lagoon in Abidjan, Ivory Coast, or the lakes formed by runoff in Antananarivo, Madagascar) is also another way to absorb contaminated water and thus to sustain “re-entry” of microorganisms in humans.

In this environmental cauldron, the release of antibiotics, intensively used for human, veterinary, and agricultural purposes, resulted in their accumulation in all freshwater, seawater, and groundwater environments worldwide [15,16]. The main concern for this release of antibiotics is related to the development of antibiotic resistance genes (ARGs) and bacteria (ARB), which reduce the therapeutic potential against pathogens, as exemplified in Africa [17,18,19,20]. This constitutes now a serious global threat to human health, causing millions of deaths each year [21], particularly in low-income countries [22].

There is thus an urgent need to better investigate relations between environmental, human, and animal microbiota mixing in surface water in the towns, to anticipate or detect the emergence of pathogens and drug resistance. The methods to conduct this survey needs to be redefined, and the places where exchanges between human and environmental microbiota occur need to be established. Knowledge of the microbial ecology of urban surface water, including the occurrence of waterborne pathogens, remains too limited [23,24]. In particular, the ubiquitous proliferation of floating plastic waste may play a new but major role in this ecology. Their role needs to be investigated owing to the crucial role biofilms that develop on their surface might play in facilitating microbial interactions and enhancing genetic exchanges [25]. Simultaneously, the release of contaminant loads (e.g., pesticides, heavy metals, pharmaceuticals, personal care products), which create new constraints for environmental microorganisms, must be analyzed. They exert selective pressures on microorganisms that may stimulate antibiotic resistance gene acquisition and spreading [26]. A revised approach for antibiotic resistance surveys that mobilize high-throughput next-generation sequencing methods [24] is thus necessary [27].

This paper illustrates the main pieces of the puzzle favoring the survival and dispersal of pathogens in urban aquatic reservoirs to open the discussion on the hot spots where genetic exchange and microbial proliferation can occur and the strategy that can be utilized for monitoring microbial communities.

## 2. Uncontrolled Urbanization Creates Large Reservoirs of Environmental/Human Composite Microorganism Communities in Surface Water Favoring Pathogen Evolution

Enteropathogens, such as enterotoxigenic *Escherichia coli* (ETEC), rotavirus, *Vibrio cholerae*, Campylobacter, and *cryptosporidium*, are major agents of diarrhea. They are good models for analyzing the propagation of pathogens in urban settings because their epidemiology is shaped by their ability to survive and to be transported from one household to another. Sustained by environmental factors, a single transient episode of contamination is often sufficient to start an epidemic, as seen during the 2011 cholera outbreak in Haiti [28]. Therefore, what are the major determinants of pathogen propagation in suburbs?

### 2.1. Environmental Factors Modulate Human Pathogen Dynamics

The growth, diversity, and dynamics of pathogenic populations are shaped by environmental factors such as rainfall, temperature, nutrient loading, UV light, water flow, pH, and availability of carbon sources [29]. A good model to assess these effects is *V. cholerae*, as its proliferation is modulated by water temperature, pH, salinity, and plankton blooms [30]. These environmental parameters strongly control both the abundance and diversity of aquatic communities, which, in turn, control pathogen proliferation. High temperatures, sunlight, and nutrient inputs favor the growth of phytoplankton and bacteria (e.g., cyanobacteria). They also control top-down relationships linking microbial communities (such as *Vibrio* sp.) with their environmental reservoir like shellfish and zooplankton [29,30,31,32]. This was well described in reservoirs in Burkina Faso where alkaline pH and phytoplankton biomass sustain the proliferation of *V. cholerae* [33]. This was also described in wastewater ponds in Australia [34], where waterborne pathogens are controlled by a range of chemical or physical factors, including salinity, pH, turbulence-induced resuspension, and macronutrients. Viruses are also concerning as they can aggregate and adsorb to particles, sustaining their persistence in surface waters.

According to the physical parameters of the media, pathogens can modulate their metabolism to survive. Microorganisms can become uncultivable but persist for a long time, which impacts the techniques and the timing available to analyze the samples. Indeed, during nutrient starvation, iron limitation, or changes in salinity and pH, *V. cholerae* survives by losing its flagellum and becoming a spore-like uncultivable bacterium [31]. *Escherichia coli*, or *Campylobacter jejuni*, can also enter the viable but noncultivable (VBNC) state (also called dormancy) with low metabolism, allowing their survival under poor environmental conditions and resuscitation when conditions become more favorable. Resuscitation windows vary from a few days to several years (e.g., *Vibrio vulnificus*, 3 days; *Vibrio fluvialis*, 6 years [35]). Similarly, bacteria from the Firmicutes family can form endospores that can survive for several years in soil, plants, or sediments and are resistant to chlorination. They can persist or be transported by environmental reservoirs [9], impacting the quality of drinking water extracted from surface water resources long after contamination.

### 2.2. Selection of Drug-Resistant Microorganism Can Occur in Surface Water

Antibiotics are spread in the environment via wastewater from farms and hospitals [10,36,37,38]. The treatment of plants and aquaculture has recently become another major contributor of antibiotics. They promote the selection of resistance genes and the development of antibiotic-resistant bacteria [39,40]. The long-term irrigation of soils with untreated wastewater leads to an accumulation of these drugs at high levels in the soil. Freshwater and marine ecosystems have become reservoirs of resistance genes and antimicrobial-resistant bacteria, which are easily transferred to human pathogens [41,42]. In this setup, the acquisition of multidrug resistance enhances the propagation and emergence of pathogens [43]. Studies conducted in Dhaka (India) illustrate this point as well; two multidrug-resistant bacteria were found that were associated with water runoff, i.e., a *Pseudomonas aeruginosa* resistant to all antibiotics except ceftazidime and an *E. coli* sensitive only to ceftazidime and cotrimoxazole [44]. In this area, *E. coli* was thus found as the causative agent in 63% of the diarrhea cases in children. However, 73% of them were resistant to at least one of the 10 antibiotics tested, and 36% were multidrug-resistant. In the same line, the emergence of multidrug-resistant “El Tor” strains in Nigeria was also related to the presence of antimicrobial drugs in the biotope [45].

Aside from pathogens, drug resistance has also been described in environmental bacteria. The level of resistance varies with the place of collection of the microorganisms as the concentration of drugs accumulated in the environment also varies [46,47]. In surface water, antibiotics can usually inhibit 25% to 76% of wild-type environmental bacteria [48]. However, when studies are conducted on river sediments, swine feces, lagoon water, liquid manure, or farmed soil, the resistance of environmental bacteria can reach 60%, 92%, 100%, and 30%, respectively [48]. For these uncultivable environmental bacteria, antibiotic resistance genes (ARGs) can be searched using metagenomics, as illustrated in tannery wastewater [49], in which various ARGs have been identified at high abundance. Over 70 types of insertion sequences were detected in each sludge sample, among which 20% were sulfonamide-resistant *sul1* genes. Class 1 integrase genes were prevalent in the whole tannery wastewater treatment plant. Tetracycline resistance genes (particularly *tet33*) were highly prevalent in anaerobic sludge but not in aerobic sludge [49]. This important finding draws attention to the role of various chemicals in the induction of drug resistance.

Aside from classic resistant pathways, some environmental bacteria can also degrade drugs through specific pathways that are not primarily involved in resistance but that belong to “the hidden resistome” [50]. This term covers all the pathways that can inhibit drug effects through a “mineralization” process of the drug. More than 90% of seawater bacteria are resistant to more than one antibiotic, and 20% are resistant to at least five [51]. Little is known about the potential effects of exogenous antibiotics on the diversity and functioning of bacterial communities in aquatic ecosystems, but ARG could be transferred to environmental bacteria. They have been suspected to shape the microbial community composition in freshwater reservoirs [52], promoting the occurrence of resistant strains (*Actinobacteria* and *Firmicutes*).

Moreover, lots of studies highlight the capacity of environmental bacteria to produce natural antibacterial substances that can be produced by marine heterotrophic bacteria, which may inhibit or kill other bacteria [53,54,55,56,57]. The antibacterial activity of two *Ludwigia* species (invasive aquatic weeds) against a series of bacteria, including pathogens, has been demonstrated [58]. The production of antibiotics as allelochemicals by phytoplankton and cyanobacteria has also been documented [59,60,61,62,63]. However, antibiotic-induced interactions among marine microorganisms have mostly been highlighted within marine biofilms [64]. These natural antimicrobials are mostly considered signaling molecules within species and not as veritable chemical weapons against other organisms [65]. Supporting this view, antibiotics are often produced at subinhibitory concentrations, as the metabolic cost of this production is relatively high. Overall, and due to this natural chemical war, many environmental bacteria have a capacity for fast evolutionary development of tolerance against antimicrobials [65].

At last, heavy metals are ubiquitous contaminants spread by runoff and sewage water into aquatic ecosystems, where they exert selective pressures on aquatic microbial communities. Heavy metals persist within sediment, where they increase the half-life of antibiotics [66]. Even at low concentrations, heavy metals can contribute to the emergence and spread of antimicrobial-resistant strains through coselection of genetic elements encoding both heavy metal and antibiotic resistance genes [67,68,69,70,71]. This was recently confirmed by the selection of antibiotic-resistant *enterococci* by very low heavy-metal concentrations [72].

Environmental bacteria are thus an unlimited source of resistance genes that might be transferred to pathogenic organisms. This transfer of genes from environmental to pathogenic bacteria has already been demonstrated. High densities of bacteriophages are released into water via defecation [73], while for soil bacteriophages, lysogeny is the prevalent reproductive strategy supporting horizontal gene transfer via transduction [74]. In *Salmonella enterica*, *Staphylococcus aureus*, *E. coli* O157:H7, or *Vibrio cholerae*, phages promote the secretion of toxins, effectors or regulatory proteins, adhesins, and serum resistance factors [75]. In this context, the domestic use of raw water might expose the human microbiota to new enzymatic structures from environmental microorganisms through lateral transmission. This transfer was already described in humans [76], sustained by integrons [77,78].

### 2.3. Urban Farming and Untreated Water Consumption Are Sources of Pathogen Contamination for Humans

Enteric pathogens are not native to freshwater. They are imported from various sources, including animal and human feces [9]. Informal settlement areas around water surfaces generate high runoff and significant fecal pollution with a range of 4 to 8 log orders of bacteria [5]. When surface water is used for watering, urban farming and consumption of vegetables such as lettuce are thus major factors of human contamination [6,7,79]. The city of Kumasi in Ghana where reuse of wastewater has become usual is a good study case as water was found contaminated by fecal bacteria, such as *Salmonella*, viruses, and *Cryptosporidium* [80]. In this city, the prevalence of intestinal protozoan infection in primary school children reached 43% (mainly *Giardia lamblia*, *Entamoeba histolytica*/*E. dispar*, and *Cryptosporidium parvum*) [81]. This study also illustrated that the assessment of enteric pathogens in feces of children living in an area can be used as a sentinel marker of contamination of water. The mass distribution of anti-parasite drugs in schools should thus be preceded by stool collection and examination. The prevalence of each pathogen could be investigated, as well as their burden illustrated, by the speed of recontamination after treatment. Molecular techniques such as multiplex Q-PCR could be implemented for automatic analysis.

## 3. Where to Survey the Emergence of Pathogens?

Surveys conducted in dispensaries with humans experiencing diarrhea are key strategies to detect new pathogens. Indeed, new pathogens will usually cause symptoms due to a lack of immunity. Systematic sampling of stools could be easily implemented in children from poor suburbs of the city, as well as attending dispensaries with diarrhea. However, the size of sampling should be large enough to allow detection of rare pathogens. This last point was demonstrated by studies designed to monitor variations of pathogens related to public health interventions [82,83].

Studies could also be conducted in the environment, but sampling sites must be carefully defined. Surface water should be a key area for analysis [84,85,86,87,88]. However, due to large variations in the concentration of microorganisms, studies conducted in environmental water bodies are not sufficiently sensitive for detecting the circulation of pathogens before an epidemic. The collection of water in distribution networks is sometimes more efficient because it provides information on the contamination of the water resource from its source to the tap, including the pipes, which are usually good supports for biofilms. Network contaminations can be due to old pipes allowing the diffusion of sewage water to pipes, providing drinkable water. This is frequent when the landscape is complex, with, for example, important elevation changes, such as in Antananarivo in Madagascar [84]. This strategy is frequently used by public water supply companies, as in Antananarivo (Bastaraud et al. *in press*). Analyses conducted on steels can reveal abundant contamination with highly resistant strains sometimes harboring specific pathways involving glutathione metabolism, the SoxRS-, OxyR-, or RpoS-system [85].

As previously described, interactions between microorganisms in biofilms are also important for genetic exchange, collaborative survival in water, propagation of pathogens, or protection against adverse environmental conditions. The co-adhesion of *Pseudomonas aeruginosa* and *E. coli* O157:H7 was, for example, demonstrated to modify growth rates when bacteria are subjected to various osmotic pressures, temperatures, heavy metal concentrations, or salt stress [86,87]. Biofilms also promote the acquisition of virulence [88,89], and identification of the places to sample is thus crucial. Environment microorganisms and macroorganisms can also host pathogens and promote biofilms. For example, *Legionella* spp. can survive in free-living amoebas (e.g., *Naegleria* spp. and *Acanthamoeba* spp.) and benefit from their protection against desiccation, elevated temperature, and disinfectants [9]. The role of aquatic vertebrates and arthropods remains poorly investigated, but they may trigger interactions through biofilm formation on their shells. Aquatic vertebrates also promote the large-scale dispersion of microbes, thus sustaining the survival and even proliferation of rare microorganisms [90]. Certain bivalves, such as the invasive zebra mussel (*Dreissena polymorpha*), can host various bacterial and protozoan pathogens. Indeed, *Vibrio cholerae* can attach to a range of aquatic organisms, including shellfish, plants, algae, and zooplankton, in salty environments and persist for long periods [29], triggering intermittent outbreaks in human communities.

Finally, garbage is also an important site for complex microbial community development. In West Africa, the coastal lagoon network is particularly developed from Sierra Leone to Angola. The main towns of these countries are settled along these lagoons with runoff, garbage, and sewage discharging directly into the lagoons. The large amounts of plastics that can be seen floating on the surface of these lagoons demonstrate these impacts. Indeed, millions of tons of plastics are rejected in aquatic ecosystems yearly [60], with more than 4.4 million originating from Africa in 2010 [91]. Whatever the origin of plastic debris, they have a lifespan of decades, if not centuries [92]. Plastics float and disperse due to winds and currents, and can travel over very long distances. During these travels, they will be degraded and fragmented, creating micro- and nanoparticles. The impacts of the degradation of plastic debris on biodiversity are still poorly known [93,94,95], but plastics can be easily colonized by microorganisms and biofilms [96,97]. They can play a major role in the transfer of organisms from one place to another [98,99,100,101,102,103]. Phytoplankton and zooplankton can develop on these supports and promote the production of bacteria and viruses, which are subsequently locally diverted to the water column [67]. Water pieces locked in floating bottles or plastic containers are also well adapted for colonization by microorganisms. Bacteria living on plastics, organic particles, and surrounding seawaters have been described [68]. The spreading of fish pathogens has also recently been described [104]. Plastic-specific bacteria constitute a distinct set of microorganisms that are very different from the surrounding water [105,106,107,108], with the quantity of bacteria 500 times more elevated than in the surrounding water [109]. Human pathogens such as *Vibrio cholerae* and *E. coli* can also stick to such supports [110,111]. Kirstein et al. (2016) reported that 13% of the debris they studied (by cultivation) contained *Vibrio* spp. We also confirmed this finding in the Abidjan lagoon (Vakou et al. in preparation). Conversely, Debroas et al. [112] performed metagenomic analysis and recorded only a 0.14% prevalence of *Vibrio* on the material they studied. They pointed out the opportunism of these bacteria (‘hitchhikers’) that use debris as a simple support unlike the vast majority of other taxa that showed a true affinity (biodegradation) for the polymers. In the marine environment (North Sea), Oberbeckman et al. [108] obtained comparable results and indicated that different communities comprising both prokaryotes and eukaryotes developed according to the nature of the plastic (polymer composition), confirming the observations of Carpenter and Smith (1972) [113]. It was suggested that organic aggregates may facilitate the survival of aquatic pathogens by providing both a ‘refuge’ [114] and a ‘resource-rich microhabitat’ [115].

Overall, and regardless of their size, floating plastics behave as ideal bioreactors where bacteria settle and proliferate. To extensively investigate the potentially pathogenic bacterial strains, present in urban tropical surface water masses, plastics must be sampled, and their biofilms must be imperatively studied in parallel with the classically monitored microbial communities of the water column and sediments. Metagenomic will be the best approach to study complex microbiota on these supports.

## 4. How to Identify Complex Microbiota

### 4.1. Different Technical Approaches

To survey the emergence of pathogens in environmental water, the detection of bacteria, viruses, and parasites is required. This detection refers to two major problems: How and when?

Classic bacteriological techniques can be used to detect contamination of surface water, i.e., (i) the fecal bacteria index (FIB), which consists of measurements of *Escherichia coli*, intestinal *enterococci*, and *Clostridium perfringens* densities [9]; (ii) microbial source tracking (MST), which can confirm fecal contamination; and (iii) host-specific fecal DNA markers, which can be used to differentiate human and nonhuman sources [116]. However, these methods do not help forecast the emergence of new pathogens. In the same line, the emergence of antibiotic resistance can be first detected by phenotypic methods using antibiotics in culture. Proteins involved in drug resistance can be detected from isolated colonies by MALDI-TOF MS-MS. However, this cannot be applied to noncultivable microorganisms, like most environmental bacteria. Detection of genetic structures coding for “drug resistance” means that associated proteins are thus required, which can be achieved by metagenomic and whole genome sequencing.

By analyzing DNA (i.e., with next-generation sequencing (NGS) techniques), complex microbial populations can be analyzed in the same round. This leads to microbial diversity, genetic adaptation, or interactions data [117,118]. However, there are still many technical pitfalls for deploying these innovative methods in low-income countries: (i) the preservation of environmental RNA or the extraction of high-molecular-weight DNA is not straightforward [119], (ii) cDNA analysis (i.e., for viral RNA), which necessitates biomass collection, total RNA extraction, storage, and cDNA synthesis [120], (iii) the maintaining of a quantitative estimation to avoid losing the representativity of the sampling.

### 4.2. How to Use Metagenomics?

Most environmental microorganisms are uncultivable, and culture-based methods cannot be used. Analysis of DNA is however always possible, allowing direct identification of microorganisms in sludge or in wastewater [121]. These studies are permitted by the drastic decrease in the price of next-generation sequencing (NGS), which is as low as 100 euros per bacterial whole genome. Metagenomic analysis consists of DNA sequencing, (i) without targeting specific DNA sequences (whole genome sequencing (WGS)), or (ii) targeting the 16S gene for bacteria and 18S gene for eukaryotes. Specific genes can also be targeted as those involved in drug resistance.

NGS generates a large number of small sequences (usually 100–200 base pairs), which must be compared to an international database. The quality and completeness of this database are thus key elements. For 16S RNA identification, the good quality of the international databases leads to reliable results. However, for environmental or new pathogen species, databases are still limited, impairing the identification. Similarly, whole-genome sequencing can be used to explore genetic diversity and to identify new enzymatic structures from the environment. However, this approach requires fully annotated genomes or, at least, previous identification of the specific genetic signatures of drug resistance “cassettes” [122]. Finally, the quantity and quality of the DNA available, as well as the deepness of sequencing (i.e., the number of times a single base will be sequenced) will also limit the sensitivity of this approach, especially for complex metagenomes mixed with DNA from contaminants issued from water treatment plants. The deeper the analysis, the more expensive it is.

For environmental biotopes, a few hundred publications are already available using this approach, especially in fresh water contexts [118]. In these samples, some species were described as cosmopolitan, i.e., Actinobacteria [123], but many new species (especially viruses) were described [29,124]. This diversity concerns microbial eukaryotes as well (nematodes, protists, fungi), found in marine sediments, which can play pivotal roles in maintaining ecosystem function [125]. Archaea have also been described in sediments [126,127]. Investigations in ponds [128] and oceans [129] have also highlighted an unknown and rapid turnover of viruses and microorganisms. New viruses (nucleocytoplasmic large DNA viruses) were discovered to play a crucial ecological role in the sea by accelerating the turnover of their unicellular hosts or by causing diseases in animals. Their abundance was extremely high, with up to 10^4^–10^5^ genomes mL^−1^ in the photic zone.

They also used 18S rDNA to investigate eukaryotic microbiota in freshwater [130] and seawater samples [131]. Cytochrome oxidase 1 barcodes were used to identify macroinvertebrates in benthic samples [132,133]. The shotgun sequencing approach was more recently used to investigate microbial and viral diversity in sea water [134,135]. Whole genome sequencing (WGS) was also used to reveal new structures involved in metabolic pathways or mutant proteins [136,137,138,139,140], or nucleocytoplasmic large DNA viruses [141].

### 4.3. Technical Approaches and Pitfalls in Genomic Analysis

The first step of metagenomic approaches is to obtain good-quality DNA or cDNA, which needs (i) biomass collection, (ii) DNA/RNA preservation, (iii) total RNA extraction, and cDNA synthesis [119]. Preservation of RNA from environmental biotopes or extraction of high-molecular-weight DNA are not straightforward [142] and require quantitative approaches, to ensure the representativity of the material [129]. The high-molecular-weight DNA will be used as a template for sequencing libraries of different sizes (200/300 bp to 3/5 kb) to facilitate genome assembly. When the DNA quantity is not sufficient in the sample, new strategies of whole genome amplification can be used. However, the quality of the data obtained in this way is usually very low. Up to 90% of the sequences generated can be from the self-amplification of primers. At the same time, under-represented DNA cannot be fully sequenced according to the deepness of the sequencing step, and the choice of the polymerase used for amplification will impact the quality and representativity of data [143].

Using the WGS approach, reconstitution of whole genomes from rough data obtained from a mix of organisms such as biofilm is challenging, as a lot of contigs will contain sequences not matching the genome guides [144]. For this analysis step, several software needed to be applied, i.e., sequence trimmers, duplicate remover, and assemblers (such as SOAPdenovo) [145,146]. Protein-coding genes (open read frames, ORFs) are predicted using a gene-finding algorithm such as MetaGeneMark [147,148,149,150,151,152,153,154,155]. Functional annotation can be performed using a BLASTP search, first against the reference genome and then against the database of nr protein sequences.

In addition to this whole genome approach, PCR/sequencing techniques targeting 16S [156] or 18S tRNA can be used to simplify the analysis. Most publications used the V3-V4 region of the rRNA gene, which contains very-well-conserved sequences bordering hypervariable regions. The high-throughput sequencing of PCR products is performed, and the results are trimmed. Trimming of the reads also requires several steps: (i) paired-end reads are filtered for quality control (using QIIME v1.7, for example [157]), (ii) tags attached to the sequences are compared against the Gold reference database to detect chimera sequences and to remove them [158], (iii) sequences with effective tags are assigned to the same operational taxonomic units (OTU) at 97% similarity using software such as UPARSE [159], and (iv) representative sequences are annotated against the small ribosomal subunit rRNA SILVA database [160]. The relative abundance of a given taxon in a biofilm is calculated as the percentage of the number of sequences assigned to this taxon divided by the total number of sequences assigned to all the taxa in the community. For sequences absent from databases, the so-called de novo strategy can be used. It consists of comparing the sequences from a dataset and then grouping the sequences by similarity into clusters that elect a consensus sequence. The consensus sequence can, in turn, be annotated by a database, defining an unknown species. Once the taxonomic assignment has been completed, the metagenomic profiles of species present in each sample will be established.

Compared to WGS, this approach offers a higher selectivity and generates a small set of data, which is easier to handle to explore species. However, the capacity of PCR to amplify the 16S gene depends on the primers used. The diversity detected is thus more limited than with WGS and the identification of new or divergent microorganisms is difficult due to the lack of accurate databases and/or adapted primers. The coverage of sequencing (i.e., the number of times a specific base is determined) will also impact the detection of variants or rare microorganisms. Preliminary studies must be conducted to determine the coverage needed to reach a plateau in the genus or species diversity detected. Some nontranscribable sequences, such as ITS, can also be used for barcoding, especially for eukaryotes [161], but this approach underestimates the diversity of the species [162] to a few key species rather than the whole genus [163].

Specific analysis of antibiotic resistance genes (ARG) can be performed in the same line as 16S using NGS. The NCBI nonredundant database (NCBInr) is used to predict their phylogenetic origin. Putative mobile genetic elements (MGEs) are then searched for and screened on the PFAM and TIGRFAMS databases [148,149]. The abundance and diversity of ARG families in the metagenomic raw sequence data sets are then analyzed by screening with the resistance-gene-specific profile hidden Markov model (HMM) with packages such as the HMMER package [150] and/or with the same data-processing workflow as for soil microbiota [151]. Predicted protein sequences can be compared to CARD [152], ARDB [153], and ResFinder databases [154] using BLASTP to ARG.

Mobile genetic elements (e.g., ICEs or transposons) are also important to detect, which can be performed using BLASTp through the ICEberg database [155]. Inspection of microsynteny (small scale of synteny) can also be performed on regions surrounding resistance genes to detect mobile elements (e.g., transposase-encoding genes) or their traces. In addition, prophages and clustered regularly interspaced short palindromic repeats (CRISPR) can be identified using PHAST and CRISPRfinder, as previously described [139]. To further assess potential ARG mobility, the assembled metagenomic contigs are aligned to plasmid genome sequences (available in the NCBI RefSeq database). Antibiotic-resistant ORFs are considered colocalized with an MGE if they shared a contig with an MGE ORF.

### 4.4. Questioning the Relevance of the Results of Metagenomic Analysis

After metagenomic analysis, questions about the relevance of the microorganism identified must be considered, as well as the differentiation of dead or living organisms [164].

When technical problems are solved to assure that the data and the sequences obtained are of good quality, the basic questions of ecology reappear. In a particular biotope, what will be the relevance of a microorganism detected in a specific site and at a precise time? What will be the turnover of the microorganisms and the risk of missing them? These questions should be addressed before determining the sampling strategy. Indeed, microbial populations seem very spatially structured in biotopes even when no physical barriers separate them. The sampling strategy can induce huge biases in the results. Diverse parameters can drive phenotypic and genotypic frequency variations in microbial communities and influence the extent and structure of microbial diversity [122,139]. The collection of representative samples thus requires careful consideration of the environmental context and a clear definition of the objectives of the sampling. The place of sampling in the biotope impacts the results, for example, in wastewater pipes [165]. Biofilms in the top and bottom of pipes were found to contain different compositions and abundances of sulfide-oxidizing and sulfate-reducing bacteria. In the same line, microbiota were found to be different in dairy lagoon wastewaters even if they were in open contact [166]. The time and season of sampling are also very important, especially in relation to climate perturbations. Local clustering was shown in the open area [167] or in ponds in relation to rainfall and storms [60] or after typhoons [168]. All environmental parameters drive phenotypic and genotypic frequency variations in microbial communities and thus control the extent and structure of microbial diversity [122,169].

Metagenomics is thus a powerful approach to explore biodiversity in environmental water. However, questions on the relevance of data still exist, and the strategy of sampling will be the most important factor leading to representativity of the results.

## 5. Conclusions

Rapid, inadequate, unplanned urbanization and high population densities are associated with poor environmental and sanitary contexts. In low-income countries, urban runoffs or open sewage channels are major sources of fecal contamination of the environment. Surface waters and urban aquatic ecosystems are thus collecting and concentrating pathogen populations, which secondarily mix with environmental microorganisms. For these pathogens, genetic drift, chemical pressure, and lateral genetic transfer facilitate acquisition of virulence, long-term survival, and multidrug resistance genes. This contributes to the emergence or re-emergence of waterborne pathogens. Virus emergence from rural (and forest) areas like Ebola or Lassa are well documented by the mass-media, whereas no attention is paid to bacteria evolving to be highly resistant in crowed suburbs of tropical cities. The use of urban surface water for bathing, farming, fishing, or as an unimproved drinking-water source means that pathogens and contaminants are discharged in water resources and mix with environmental aquatic microbiota, before coming back to humans. In the same time, between 2000 and 2010, the consumption of antibiotic drugs increased by 35% [22] with a huge increase in drug resistance and deaths. The emergency is to preserve the efficacy of existing drugs for future generations [170]. Modern analytical processes, i.e., metagenomic approaches, have to be implemented to comprehensively study this complex biotope as many of these microorganisms are not cultivable and escape classical microbiology approaches. In the same line, the places to carry out sampling to study bacteria drift cannot be simply water and sediments as classically done but must take into account new habitats associated with floating macroaggregates, especially plastics. The pervasiveness of plastics is said to be the ultimate geological proxy for characterizing our new Anthropocene era. The ubiquity of plastics reshapes aquatic habitats, deeply impacting microbial communities and their interactions. Resistance gene transfer pathways might thus be noticeably enhanced, with huge potential epidemiological consequences.
